# Contribution of crossing over to genetic variance in maize and wheat populations

**DOI:** 10.1002/tpg2.20552

**Published:** 2025-01-08

**Authors:** Rex Bernardo

**Affiliations:** ^1^ Department of Agronomy and Plant Genetics University of Minnesota Saint Paul Minnesota USA

## Abstract

Crossing over breaks linkages and leads to a wider array of allele combinations. My objective was to assess the contribution of crossing over to genetic variance (*V*_G_) in maize (*Zea mays* L.) and wheat (*Triticum aestivum* L.). The contribution of crossing over to *V*_G_ (denoted by *P*_CO_) was assessed by calculating *V*_G_ without crossing over from the sums of marker effects on each chromosome and by estimating *V*_G_ with crossing over from simulated doubled haploids that arise from meiosis. For maize yield, crossing over had positive contributions of *P*_CO_ = 7% and 16% in two populations but it strongly decreased *V*_G_ (*P*_CO_ = −74% to −25%) in five other populations. The mean *P*_CO_ was negative for moisture, test weight, plant height, and ear height. In wheat, the *P*_CO_ values were all negative for five traits in the Louise/Penawawa population but were all positive for three traits in the Seri/Babax population. Negative *P*_CO_ values were attributed to large differences between the sum of allelic effects on a homolog inherited from one parent and the sum of effects on the homolog from the other parent. Although crossing over most often decreased *V*_G_, the best simulated line (out of 10,000) with crossing over was usually superior to the best line without crossing over. Breeding progress will therefore continue to rely on finding individuals with increasingly rare, favorable crossovers amidst individuals with crossovers that are mostly unfavorable.

## Core Ideas

- Crossing over generally decreased the genetic variance (*V*_G_) in maize and wheat populations.
- This decrease in *V*_G_ was largely due to the disruption of favorable blocks of alleles inherited from one parent.
- The very best line (out of 10,000) is expected to be superior with crossing over than without crossing over.

**Abbreviations**
QTL: quantitative trait loci
RR‐BLUP: ridge regression‐best linear unbiased prediction
SNP: single nucleotide polymorphism

## 1 INTRODUCTION

Genetic improvement requires genetic variation. In plant breeding, genetic variation is typically generated by crossing two parents and allowing meiosis and fertilization to generate progeny that have gene combinations absent in either parent. To illustrate, if two wheat (*Triticum aestivum* L.) parents have the *AABB* and *aabb* genotypes, genetic improvement at these two loci relies on being able to generate homozygous lines with the *AAbb* and *aaBB* genotypes and to assess whether these lines are superior to the parents.

Nonparental genotypes arise from two mechanisms: independent assortment and crossing over. If the *A* and *B* loci are on different chromosomes, independent assortment of chromosomes during meiosis will lead to all four possible gametes (*AB*, *Ab*, *aB*, and *ab*). If the *A* and *B* loci are close to each other on the same chromosome, such linkage will lead only to the parental gametes (*AB* and *ab* in this example) and the nonparental or recombinant gametes (*Ab* and *aB*) are formed only if crossing over occurs. Such crossing over is evidenced by the formation of chiasma during the first meiotic division, with 1–4 chiasmata typically being formed between homologous chromosomes (Crismani & Mercier, 2012; Lian et al., 2023).

The regulation and modulation of crossing over is an active area of research (Blary & Jenczewski, 2019; Mieulet et al., 2018; Pelé et al., 2017), with the supposition that increased recombination will lead to more allele combinations that, in turn, would lead to a larger genetic variance (*V*_G_). But for quantitative traits, the contribution of crossing over to *V*_G_ has not been reported. In other words, in a cross between two parental lines, what proportion of *V*_G_ is due to allele combinations that arise from crossing over? My objective in this study was to assess the extent to which crossing over contributes to *V*_G_ for yield and agronomic traits in maize (*Zea mays* L.) and wheat populations.

## 2 MATERIALS AND METHODS

### 2.1 Maize and wheat populations and genomewide marker effects

Genomewide marker effects were calculated for nine biparental populations that have been previously described. These populations included seven maize breeding populations from AgReliant Genetics (Krchov et al., 2015) and the Louise/Penawawa (Carter et al., 2011, 2012) and Seri/Babax (Liu et al., 2019) wheat populations.

The seven maize populations comprised different combinations between four biparental crosses (A/B, C/B, C/D, and E/F) and three testers (T1, T2, and T3). The six parental inbreds all belonged to the Iowa Stiff Stalk Synthetic heterotic group. The marker similarity between the parental inbreds was 0.69 for A/B, 0.78 for C/B, 0.53 for C/D, and 0.69 for E/F (Krchov et al., 2015). Each biparental cross had 130–242 doubled haploids (Table 1). Testcrosses of the doubled haploids were evaluated in 18 environments (year–location combinations, in 2008–2012, in the US Corn Belt) for yield (Mg ha^−1^), moisture (g H_2_O kg^−1^), test weight (kg hL^−1^), plant height (cm), and ear height (cm) (Krchov et al., 2015). Data for 3072 single nucleotide polymorphism (SNP) markers were filtered via SNP‐QC software (Bernardo, 2020a) to discard markers that had >20% missing data, a minor allele frequency <0.10, or a linkage disequilibrium of *r* ^2^ > 0.9025 (*r* = |0.95|) with other marker loci. After filtering, the number of SNP loci in each maize population ranged from 185 to 318 (Table 1). A linkage map for each maize population was constructed via QTL IciMapping version 4.2 software (Meng et al., 2015). Genomewide marker effects were calculated via ridge regression‐best linear unbiased prediction (RR‐BLUP) as implemented in RRBLUP software (Bernardo, 2020a). Genomewide marker effects for the maize populations, as well as for the wheat populations described below, are provided as supporting information.

**TABLE 1:** Number of lines (*N*), number of markers (*N*_M_), and predictive ability in seven maize populations and in the Louise/Penawawa and Seri/Babax wheat populations.

			Predictive ability								
Population	*N*	*N*_M_	Yield	Moisture	Test weight	Plant height	Ear height	Solvent retention	Milling score	Cookie diameter	Grain number
A/B (T1)	134	206	0.76	0.77	0.66	0.70	0.73				
A/B (T2)	130	205	0.61	0.76	0.76	0.46	0.50				
C/B (T1)	242	185	0.56	0.64	0.28	0.31	0.33				
C/B (T2)	240	188	0.49	0.76	0.78	0.46	0.50				
C/D (T1)	140	318	0.72	0.81	0.77	0.67	0.61				
E/F (T1)	140	187	0.39	0.81	0.44	0.56	0.63				
E/F (T3)	141	186	0.30	0.67	0.55						
Louise/Penawawa	187	301	0.47		0.60			0.64	0.65	0.49	
Seri/Babax	155	525	0.47			0.20					0.56

The Louise/Penawawa population comprised 187 recombinant inbreds evaluated in four environments in Washington and Idaho in 2007–2008 for yield (Mg ha^−1^), test weight (kg hL^−1^), cookie diameter (cm), milling score, and flour solvent retention capacity for water (solvent retention, for short; Carter et al., 2012). The Seri/Babax population comprised 155 recombinant inbreds evaluated in Obregon, Mexico, in 2004–2009 for yield (Mg ha^−1^), 1000‐grain weight (g), grain number per plant, heading date (d), and plant height (cm) (Liu et al., 2019). Procedures for quality control of marker data in these two wheat populations were described by Bernardo (2021). The RR‐BLUP genomewide marker effects were those previously calculated by Bernardo (2021) for 301 SNP and simple sequence repeat loci in the Louise/Penawawa population and for 525 SNP loci in the Seri/Babax population. Shrinkage factors specific to the A, B, and D subgenomes of wheat were used in RR‐BLUP. The Louise/Penawawa linkage map was obtained from Ru and Bernardo (2019), whereas the Seri/Babax linkage map was obtained from Liu et al. (2019).

Results for 1000‐grain weight and heading date in the Seri/Babax population were excluded because of their low predictive ability (<0.20) from delete‐one cross‐validation. The predictive ability for the remaining 41 population‐trait combinations ranged from 0.20 to 0.81 (Table 1).

### 2.2 Simulation of doubled haploids with and without crossing over

For each population, 10,000 random gametes were produced by simulating meiosis in the F_1_ between the two parents. The number of crossover events within each chromosome was simulated according to a truncated Poisson distribution (Bernardo, 2014), with a minimum of zero and a maximum of four crossovers per homologous pair. An ad hoc Fortran program was used for the simulations. A minimum of one chiasma (i.e., obligatory crossover) is required for the proper disjunction of homologous chromosomes during meiosis (Wang et al., 2015), yet empirical marker data have indicated no crossover events on one or more chromosomes in maize populations (Smith et al., 2008). These two seeming contradictions are resolved when we consider that an obligatory crossover may occur next to a monomorphic locus, making the obligatory crossover undetectable from marker data.

The mean number of crossovers per chromosome pair was 1.0 per 100 cM, with the number of cM per chromosome being from the respective linkage maps. Locations of the crossovers on a chromosome followed a uniform distribution, with the restriction that crossovers were at least 5 cM apart to account for some level of interference (Bernardo, 2014). A doubled haploid was formed by doubling the chromosomes in each random simulated gamete. With *N*_M_ being the number of markers in the population, the genotypic values of the 10,000 doubled haploids in each population were predicted as **Xg**, where **X** was a 10,000 × *N*_M_ incidence matrix of SNP genotypes and **g** was an *N*_M_ × 1 vector of SNP marker effects for the trait.

**Core Ideas**

- Crossing over generally decreased the genetic variance (*V*_G_) in maize and wheat populations.
- This decrease in *V*_G_ was largely due to the disruption of favorable blocks of alleles inherited from one parent.
- The very best line (out of 10,000) is expected to be superior with crossing over than without crossing over.

A separate set of 10,000 doubled haploids was simulated within each population under the restriction that no crossover events occurred during meiosis. Genotypic values were calculated as described above.

### 2.3 Contribution of crossing over to genetic variance

The *V*_G_ was defined as the genetic variance expressed among a group of progeny (doubled haploids), rather than as the sum of different types of genetic variances (additive, dominance, and epistatic variances) relative to a random‐mating population without inbreeding (Bernardo, 2020b). For each of the seven maize populations and two wheat populations, the mean was calculated for the 10,000 doubled haploids simulated with crossing over and the 10,000 doubled haploids simulated without crossing over. The *V*_G_ was estimated as the variance among the genotypic values of the 10,000 doubled haploids simulated with crossing over (Bernardo, 2014; Mohammadi et al., 2015). The *V*_G_ was also calculated analytically from the marker effects (Osthushenrich et al., 2017). The *V*_G_ estimated from simulated progeny and *V*_G_ calculated analytically were close in value (results not shown), as has been previously found (Osthushenrich et al., 2017). The *V*_G_ values calculated from the simulated doubled haploids were used herein because the simulations allowed an upper limit on the number of crossovers per chromosome, whereas the analytical approach did not.

The *V*_G_ without crossing over, which was denoted by *V*_G(NCO)_, was calculated from the sums of marker effects on each chromosome. The sum of the effects of the alleles from the first parent was denoted by *c*_i_ for the *i*th chromosome. Because the allelic effects at a given locus summed to zero, the sum of the effects of the alleles from the second parent was equal to −*c*_i_. With the chromosomes being independent and the mean values of the homologs being *c*_i_ + (−*c*_i_) = 0, *V*_G(NCO)_ was equal to ∑(*c*_i_)^2^. The percentage contribution of crossing over to *V*_G_ was denoted by *P*_CO_ and was estimated as *P*_CO_ = 100[*V*_G_ − *V*_G(NCO)_]/*V*_G_. Although *V*_G(NCO)_ was calculated analytically, the simulations without crossing over were still needed because of the non‐normal distributions without crossing over, as described later.

Although *V*_G_ is the total genetic variance and *V*_G(NCO)_ is the genetic variance without crossing over, [*V*_G_ − *V*_G(NCO)_] should not be considered as the genetic variance due to independent assortment. The reason for this caveat is that independent assortment is due to (1) loci that are on different chromosomes and (2) loci that are far enough on the same chromosome, to the extent that crossing over occurs frequently enough for such loci to assort independently. The *V*_G(NCO)_ captures the genetic variance due to segregation of loci on different chromosomes but it does not capture the genetic variance due to independent assortment of loci found on the same chromosome.

For each trait, percentile values for the best 5% and best 1% of the population were determined according to whether lower trait values (moisture, plant height, and ear height) or higher trait values (yield and the remaining traits) are desired. Percentile values were determined by sorting the genotypic values of the 10,000 simulated doubled haploids. Four ratios were calculated: between the (1) square root of *V*_G(NCO)_ and the square root of *V*_G_, with the ratio denoted by *R*_SD_; (2) the best fifth‐percentile value without crossing over and the best fifth‐percentile value with crossing over, with the ratio denoted by *R*_5%_; (3) the best first‐percentile value without crossing over and the best first‐percentile value with crossing over, with the ratio denoted by *R*_1%_; and (4) value of the best line without crossing over and the value of the best line with crossing over, with the ratio denoted by *R*_Best_. As indicated in the Discussion section, any inequality among *R*_SD_, *R*_5%_, and *R*_1%_ indicated a deviation from a normal distribution. Coefficients of skewness and excess kurtosis were calculated for both sets of 10,000 doubled haploids, and the statistical significance (*p* = 0.05) of the coefficients was assessed via standard *z*‐tests.

## 3 RESULTS

Crossing over most often decreased the *V*_G_ in the seven maize populations. For yield, crossing over had positive contributions of *P*_CO_ = 7% in the C/B (T1) maize population and *P*_CO_ = 16% in the E/F (T1) population (Table 2). But in the five other maize populations, crossing over decreased *V*_G_ by 74% (i.e., *P*_CO_ = −74%) in A/B (T1) to 25% in A/B (T2). Across the seven maize populations, the mean contribution of crossing over to *V*_G_ was negative (mean *P*_CO_ = −28%).

**TABLE 2:** Contribution (%) of crossing over to genetic variance (*P*_CO_), ratio between genetic standard deviation with and without crossovers (*R*_SD_), ratios between percentile values of best 5% (*R*_5%_) and 1% (*R*_1%_) of the doubled haploids with and without crossovers, ratio between the values of the best doubled haploids (*R*_Best_) without and with crossovers, and excess kurtosis among doubled haploids simulated for seven maize populations.

							Excess kurtosis	
Trait	Population	*P*_CO_	*R*_SD_	*R*_5%_	*R*_5%_	*R*_Best_	No crossovers	With crossovers
Yield	A/B (T1)	−74	1.32	1.37	1.25	1.01	−11.0	−4.5
	A/B (T2)	−25	1.12	1.14	1.10	0.72	−9.0	−3.1
	C/B (T1)	7	0.97	0.97	0.95	0.73	−8.6	−3.0
	C/B (T2)	−47	1.21	1.21	1.13	0.89	−11.0	−4.0
	C/D (T1)	−33	1.15	1.21	1.09	0.82	−10.5	−4.6
	E/F (T1)	16	0.92	0.94	0.86	0.67	−10.5	−2.6
	E/F (T3)	−37	1.17	1.18	1.15	0.85	−7.6	−3.8
	Mean	−28	1.12	1.15	1.08	0.81		
Moisture	A/B (T1)	−34	1.16	1.15	1.02	0.83	−14.4	−4.1
	A/B (T2)	−95	1.40	1.43	1.25	0.95	−15.5	−6.8
	C/B (T1)	−67	1.29	1.33	1.24	0.99	−11.9	−6.2
	C/B (T2)	−16	1.08	1.08	1.03	0.76	−9.7	−2.9
	C/D (T1)	−58	1.26	1.25	1.14	0.82	−14.7	−4.6
	E/F (T1)	−41	1.19	1.16	1.04	0.76	−14.7	−3.8
	E/F (T3)	−15	1.07	1.07	1.06	0.66	−8.3	−2.6
	Mean	−47	1.21	1.21	1.11	0.82		
Test weight	A/B (T1)	−92	1.39	1.37	1.31	1.02	−10.0	−4.7
	A/B (T2)	−93	1.39	1.45	1.25	1.01	−13.6	−5.8
	C/B (T1)	20	0.90	0.88	0.82	0.63	−12.6	NS
	C/B (T2)	−16	1.08	1.10	1.03	0.74	−11.4	−5.1
	C/D (T1)	−44	1.20	1.20	1.12	0.81	−13.5	−4.2
	E/F (T1)	21	0.89	0.89	0.87	0.65	−9.8	NS
	E/F (T3)	13	0.93	0.94	0.93	0.59	−5.7	NS
	Mean	−27	1.11	1.12	1.05	0.78		
Plant height	A/B (T1)	−65	1.29	1.26	1.20	1.02	−14.6	−9.0
	A/B (T2)	−50	1.23	1.26	1.09	0.92	−13.9	−5.2
	C/B (T1)	22	0.88	0.86	0.87	0.75	−6.2	−3.5
	C/B (T2)	23	0.88	0.88	0.85	0.63	−8.5	NS
	C/D (T1)	−7	1.03	0.99	0.90	0.61	−20.1	−2.3
	E/F (T1)	28	0.85	0.86	0.81	0.57	−10.5	−3.5
	Mean	−8	1.03	1.02	0.95	0.75		
Ear height	A/B (T1)	−47	1.21	1.21	1.17	0.80	−10.2	−4.4
	A/B (T2)	−62	1.27	1.27	1.16	1.01	−14.7	−6.0
	C/B (T1)	−19	1.09	1.08	1.05	0.80	−9.3	−3.3
	C/B (T2)	−54	1.24	1.32	1.12	0.88	−13.3	−4.6
	C/D (T1)	−59	1.26	1.18	1.07	0.84	−15.8	−5.1
	E/F (T1)	9	0.95	0.94	0.87	0.61	−16.7	−3.5
	Mean	−38	1.17	1.17	1.07	0.82		

*Note*: NS indicates nonsignificance at *p* = 0.05; all other excess kurtosis coefficients were significant.

The *P*_CO_ values for the seven maize populations were all negative for moisture, ranging from −95% in A/B (T2) to −15% in E/F (T3) and with a mean of −47% (Table 2). The *P*_CO_ values ranged from −93% to 21% and had a mean of −27% for test weight; from −65% to 28% with a mean of −8% for plant height; and from −62% to 9% with a mean of −38% for ear height.

Crossing over decreased *V*_G_ for all five traits in the Louise/Penawawa wheat population, with *P*_CO_ values ranging from −85% for milling score to −10% for solvent retention (Table 3). In contrast, crossing over had positive contributions to *V*_G_ for all three traits in the Seri/Babax wheat population, with *P*_CO_ values ranging from 8% for yield to 66% for plant height.

**TABLE 3:** Contribution (%) of crossing over to genetic variance (*P*_CO_), ratio between genetic standard deviation with and without crossovers (*R*_SD_), ratios between percentile values of best 5% (*R*_5%_) and 1% (*R*_1%_) of the doubled haploids with and without crossovers, ratio between the values of the best doubled haploids (*R*_Best_) without and with crossovers, and excess kurtosis among doubled haploids simulated for two wheat populations.

							Kurtosis	
Population	Trait	*P*_CO_	*R*_SD_	*R*_5%_	*R*_1%_	*R*_Best_	No crossovers	With crossovers
Louise/Penawawa	Yield	−36	1.16	1.16	1.13	0.93	−7.4	−2.9
	Test weight	−26	1.12	1.11	1.09	1.08	−9.3	−7.2
	Milling score	−85	1.36	1.33	1.21	0.86	−19.2	−7.5
	Cookie diameter	−17	1.08	1.04	0.95	0.77	−15.1	NS
	Solvent retention	−10	1.05	1.06	0.96	0.89	−10.7	NS
Seri/Babax	Yield	8	0.96	0.98	0.93	0.74	−9.6	−4.3
	Grain number	20	0.89	0.90	0.87	0.80	−7.9	−2.2
	Plant height	66	0.59	0.58	0.56	0.42	−9.0	NS

*Note*: NS indicates nonsignificance at *p* = 0.05; all other excess kurtosis coefficients were significant.

The presence or absence of crossing over did not lead to any significant differences (*p* = 0.05) in the population mean for any trait in the maize and wheat crosses. No significant skewness was detected for any trait in any population, either with or without crossing over.

The ratio between the genetic standard deviation without crossing over and with crossing over (ratio denoted by *R*_SD_) ranged from 0.85 to 1.40 across the different traits in the seven maize populations (Table 2). In the two wheat populations, the *R*_SD_ values ranged from 0.59 to 1.36 (Table 3). The ratio between the percentile values of the best 5% of doubled haploids, without crossing over versus with crossing over (ratio denoted by *R*_5%_), ranged from 0.86 to 1.45 in the maize populations (Table 2) and from 0.58 to 1.33 in the wheat populations (Table 3). The corresponding ratios for the best 1% (*R*_1%_) ranged from 0.81 to 1.31 in the maize populations (Table 2) and from 0.56 to 1.21 in the wheat populations (Table 3). The *R*_5%_ values were consistently greater than the *R*_1%_ values, except for plant height in the C/B (T1) maize population.

The ratio between the values of the best individual without crossing over and with crossing over (*R*_Best_) ranged from 0.42 for plant height in the Seri/Babax wheat population to 1.08 for test weight in the Louise/Penawawa wheat population (Table 3). Most of the *R*_Best_ values were <1.0 in the maize populations (Table 2) and wheat populations (Table 3). The mean *R*_Best_ across the seven maize populations was 0.81 for yield, 0.82 for moisture, 0.78 for test weight, 0.75 for plant height, and 0.82 for ear height.

**FIGURE 1:**
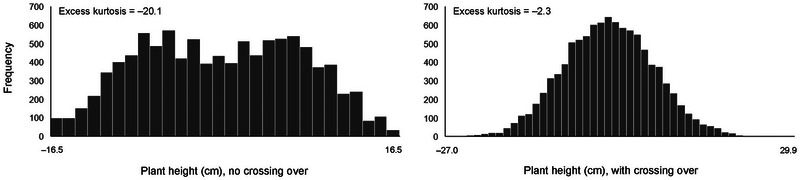
In the C/D (T1) maize population, excess kurtosis was strong for plant height (values expressed as a deviation from the mean) among doubled haploids simulated without crossing over, but kurtosis was weak with crossing over.

Excess kurtosis was always significant (*p* = 0.05) when crossing over was absent, with the excess kurtosis coefficients ranging from −20.1 for plant height in the C/D (T1) maize population (Figure 1) to −5.7 for test weight in the E/F (T3) maize population (Table 2). With crossing over, the strongest excess kurtosis was −9.0, and excess kurtosis was nonsignificant in seven out of the 41 population‐trait combinations.

## 4 DISCUSSION

### 4.1 How crossing over reduces genetic variance

The results showed that crossing over has only a minor contribution to *V*_G_ in maize and wheat, and, in most cases, crossing over actually reduces *V*_G_. A *P*_CO_ value >50% indicated that crossing over accounted for a majority of *V*_G_. The *P*_CO_ exceeded 50% only for plant height in the Seri/Babax wheat population (*P*_CO_ = 66%, Table 3). Out of the 40 remaining population‐trait combinations, 12 had *P*_CO_ values between 0% and 50% and 28 had *P*_CO_ values that were negative.

These results can be largely explained by three points. First, having a wider array of allele combinations does not necessarily lead to a larger *V*_G_. Second, crossing over is expected to increase *V*_G_ if, on any given chromosome, the favorable alleles are contributed equally by the two parents. Third, crossing over is expected to decrease *V*_G_ if, on one or more chromosomes, most of the favorable alleles are already found in one parent.

Crossing over increases the number of allele combinations, whereas any variance is increased by a preponderance of observations with extreme values. Suppose the *A* and *B* loci have equal effects, with each copy of the dominant allele having a contribution of 1 to the trait and each copy of the recessive allele having a contribution of −1. The genotypic values are 4 for *AABB*, 0 for *AAbb* and *aaBB*, and −4 for *aabb*. The variance among homozygous lines is maximized (*V*_G_ = 16) if half of the lines have the *AABB* genotype and half have the *aabb* genotype. But if the population includes lines with the extreme values as well as lines with intermediate values (*AAbb* and *aaBB*), the variance among lines decreases to *V*_G_ = 8 if each of the four homozygotes has a frequency of ¼. The *V*_G_ is therefore higher in a population that has only the *AABB* and *aabb* genotypes than in a population that includes all four homozygotes. On the other hand, *V*_G_ in this example is zero if the population includes only the *AAbb* and *aaBB* genotypes.

If crossing over is absent, a chromosome would contribute to the trait as if it were one independent locus. The sum of the marker effects on a given parental homolog then becomes important because the entire homolog is transmitted intact to an offspring. Suppose that a chromosome has eight quantitative trait loci (QTL), with each dominant allele having a contribution of 1 and each recessive allele having a contribution of −1. If one parental homolog has the *ABCDefgh* haplotype and the other parental homolog has the *abcdEFGH* haplotype, each homolog would have a value of zero and all of the resulting doubled haploids would also have a value of 0. The absence of crossing over would then lead to *V*_G_ = 0 on this chromosome. A *V*_G_ = 0 in the absence of crossing over can occur with other parental homologs as long as the sum of allelic effects is equal for the two parental homologs (e.g., *AbCdEfGh* and *aBcDeFgH*).

If crossing over occurs in the *ABCDefgh*//*abcdEFGH* F_1_, haplotypes with unequal values (e.g., *ABcdEFGH*, *abcdEFGh*, etc.) will be generated and will lead to *V*_G_ > 0 among doubled haploids. Crossing over is therefore expected to increase *V*_G_ if the sum of the effects of the alleles on one parental homolog is equal or close to the sum of the effects of the alleles on the other parental homolog. This phenomenon is akin to the hidden *V*_G_ that is released when repulsion linkage between loci is broken (Kearsey, 1985; Weir et al., 1980). In this situation, *P*_CO_ would be positive.

Conversely, crossing over is expected to decrease *V*_G_ if the sum of the effects of the alleles on one parental homolog differs substantially from the sum of the effects of the alleles on the other parental homolog. For example, if one parental homolog has the *ABCDEFGH* haplotype and the other parental homolog has the *abcdefgh* haplotype, the absence of crossing over would lead to doubled haploids with only the *AABBCCDDEEFFGG* and *aabbccddeeffgg* genotypes. These extreme‐value genotypes already lead to the maximum *V*_G_. Crossing over would lead to haplotypes with intermediate values and decrease *V*_G_ (Kearsey, 1985). In this situation, *P*_CO_ would be low or negative.

The negative *P*_CO_ values in the maize populations and in the Louise/Penawawa wheat population were consistent with large differences in the values of the homologs inherited from the two parents. For example, plant height in the A/B (T1) maize population had a strong negative *P*_CO_ of −65%. The difference between the summed marker effects of the parental homologs had a maximum of 7.68 cm for chromosome 6. In contrast, a strong positive *P*_CO_ of 66% was observed for plant height in the Seri/Babax wheat population (Table 3). The largest difference between the summed marker effects of the homolog inherited from Seri and the homolog inherited from Babax was 2.07 cm for chromosome 5A.

Because the marker effects are centered on zero, the sums of the effects of markers on the homolog from the first parent (denoted by *c*_i_ for the *i*th chromosome) are sufficient to calculate the genetic variance without crossing over, with *V*_G(NCO)_ being equal to ∑(*c*_i_)^2^ in a biparental population. A large *c*_i_ contributes to a low *P*_CO_ through its direct effect on *V*_G(NCO)_, but it does not explain all of the variation in *P*_CO_ because *P*_CO_ is also affected by *V*_G_ (with crossing over). The *c*_i_ for plant height of 7.68 cm for chromosome 6 in the A/B (T1) maize population was associated with the strongest negative *P*_CO_ (−65%) for this trait, but this *c*_i_ value was the second largest and not the largest. The largest *c*_i_ for plant height was 12.68 cm on chromosome 8 in the C/D (T1) maize population. This largest *c*_i_ was associated with only a slightly negative *P*_CO_ of −7%, (Table 2), but it was associated with the strongest excess kurtosis (Figure 1).

### 4.2 Plant breeding implications

The seven maize biparental crosses were populations undergoing selection in the AgReliant Genetics breeding program (Krchov et al., 2015), whereas the two wheat populations were not primarily created for breeding purposes. The Louise/Penawawa wheat population was created for mapping QTL for agronomic traits (Carter et al., 2011) and milling and baking quality traits (Carter et al., 2012), whereas the Seri/Babax wheat population was created for physiological and genetic analysis of drought tolerance (Olivares‐Villegas et al., 2007). The predominantly negative *P*_CO_ values in the maize populations suggest that continuous breeding had already led to the accumulation of favorable alleles on specific chromosomes of a given parent. The routine use of doubled haploid technology in maize is expected to preserve blocks of favorable alleles because developing homozygous lines after only one meiotic event in the F_1_ leads to large segments of chromosomes, or even entire chromosomes, being passed intact from parent to progeny (Smith et al., 2008).

An accumulation of favorable alleles on a given chromosome putatively arose from previous crossover events in the breeding history of the germplasm. This implies that if crossing over is currently advantageous in a given cross because it would lead to new blocks of favorable alleles, crossing over will become less advantageous in future germplasm developed from the population. Germplasm in which crossing over might be less advantageous in future breeding generations includes the Seri/Babax population, for which the *P*_CO_ values for all three traits were positive (Table 3), and the E/F (T1) maize population, for which all *P*_CO_ values were positive except for moisture (Table 2). Also, the favorable alleles are not necessarily concentrated in only one of the parents. For plant height in the C/D (T1) maize population, for example, the sum of allelic effects was lower in the homolog from the first parent for chromosomes 2, 6, 7, 8, and 9, whereas the sum was lower in the homolog from the second parent for chromosomes 1, 3, 4, 5, and 10.

Epistasis has been invoked as a reason for preserving sets of favorable alleles (He et al., 2017; Rasmusson & Phillips, 1997) but the results of this study, in which epistasis was not modeled, point to the importance of maintaining favorable linkage blocks even in the absence of epistasis. The SNP loci are biallelic, but if multiple alleles are present at QTL, a parental homolog that has all of the favorable QTL alleles in one biparental population would not necessarily have all of the favorable QTL alleles in a different biparental population. Such multiple alleles at QTL provide variation needed for further breeding progress. Multiple alleles at QTL can also be present in crosses made from three or more parents. The results herein for biparental crosses should also apply to multiparental crosses. Crossing over would increase *V*_G_ if, for each chromosome, the homologs contributed by each of the multiple parents are similar in their *c*_i_ values. Otherwise, if the homologs from multiple parents differ widely in *c*_i_, crossing over is expected to have a negative contribution to *V*_G_.

In plant breeding, a criterion more meaningful than *V*_G_ is the mean or percentile value of the best individuals in the population. For maize yield, the mean ratio between percentile values of the best 5% of lines without crossing over and with crossing over was *R*_5%_ = 1.15 (Table 2). In other words, the gains from selecting the 5% highest yielding lines would be 15% greater if crossing over was absent than if crossing over was present. The corresponding ratio for the 1% highest yielding lines had a mean of *R*_1%_ = 1.08 across the maize populations. As indicated by the *R*_Best_ values across the 41 population‐trait combinations (Tables 2 and 3), the best line out of 10,000 was most often superior when it was developed with crossing over than without crossing over. Therefore, crossing over was advantageous but it took a very large population and very stringent selection to realize this advantage (Bernardo, 2022).

As expected, the *R*_5%_ and *R*_1%_ values across different traits in the maize and wheat populations exceeded 1.0 when *P*_CO_ was negative, and they were ˂1.0 when *P*_CO_ was positive. If the distributions were normal, both *R*_5%_ and *R*_1%_ were expected to be equal to the ratio of the genetic standard deviations without and with crossing over, that is, *R*_SD_ = [*V*_G(NCO)_/*V*_G_]^½^. Non‐normality of the distributions was evidenced by the strong excess kurtosis coefficients, particularly in the absence of crossing over (Tables 2 and 3; Figure 1). Kurtosis, even with crossing over, can be attributed to having a finite rather than an infinite number of loci (Bernardo, 2022) and the high power for detecting statistically significant kurtosis given the size of the simulated populations (10,000). Comparisons based on *V*_G_, or any variance for that matter, become less meaningful when the distributions have different shapes, such as the distributions in Figure 1.

**FIGURE 2:**
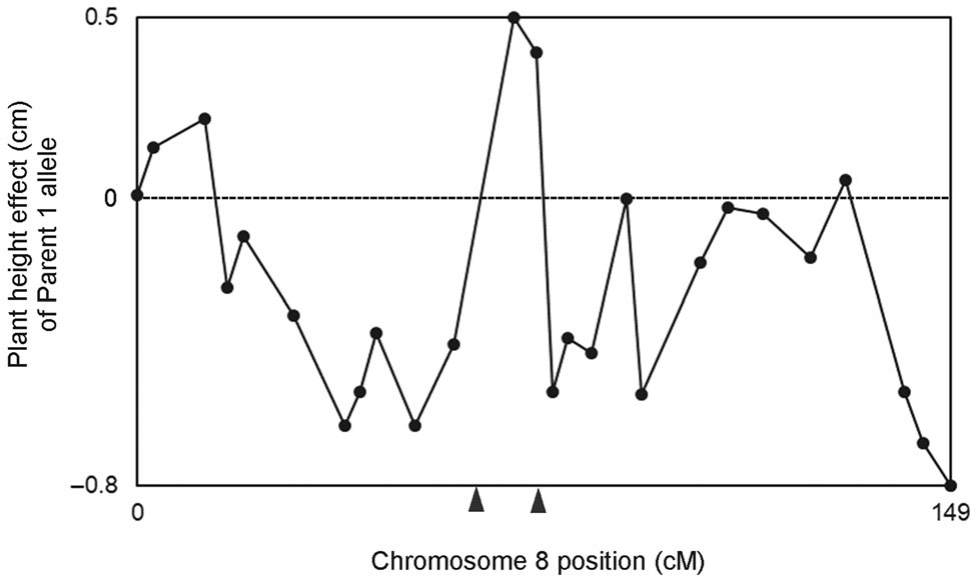
On chromosome 4 in the C/D (T1) maize population, most of the marker alleles associated with shorter plants were from the first parent. A double crossover would be favorable if it occurs at the marker intervals indicated by the two triangles on the horizontal axis.

Whereas the results indicated that crossing over decreases *V*_G_, particularly in maize, crossing over at specific genomic regions will be needed for further breeding progress. For plant height in the C/D (T1) maize population, for example, a double crossover is favorable at the marker intervals indicated by the triangles on the horizontal axis in Figure 2. Such a double crossover would be rare and would be accompanied by unfavorable crossovers elsewhere in the genome, and technologies for the artificial induction of targeted recombination are still under development (Kouranov et al., 2022). Although crossing over is expected to decrease *V*_G_ in elite breeding germplasm, further breeding progress will continue to rely on finding individuals in which (1) crossing over is absent or minimal within favorable chromosome blocks and (2) crossover events occur where they are needed to produce new blocks of favorable alleles. Such individuals are likely to be obtained not in a single generation but in multiple generations of breeding. Studies are needed on the influence of crossing over on the gains from recurrent selection, particularly with marker effects estimated from empirical data as in this current study.

## AUTHOR CONTRIBUTIONS

**Rex Bernardo**: Conceptualization; data curation; formal analysis; investigation; methodology; resources; software; visualization; writing—original draft; writing—review and editing.

## CONFLICT OF INTEREST STATEMENT

The author declares no conflicts of interest.

## DATA AVAILABLITY STATEMENT

The data are provided as supplementary material.

## Supporting information



Marker positions and genomewide marker effects for maize and wheat populations.
